# Real-Time and Rapid Respiratory Response of the Soil Microbiome to Moisture Shifts

**DOI:** 10.3390/microorganisms11112630

**Published:** 2023-10-26

**Authors:** Montana L. Smith, Karl K. Weitz, Allison M. Thompson, Janet K. Jansson, Kirsten S. Hofmockel, Mary S. Lipton

**Affiliations:** Pacific Northwest National Laboratory, Richland, WA 99354, USA; montana.smith@pnnl.gov (M.L.S.); karl.weitz@pnnl.gov (K.K.W.); janet.jansson@pnnl.gov (J.K.J.); kirsten.hofmockel@pnnl.gov (K.S.H.)

**Keywords:** soil, microbiomes, drought, respiration, Birch Effect, molecular mechanisms

## Abstract

Microbial response to changing environmental factors influences the fate of soil organic carbon, and drought has been shown to affect microbial metabolism and respiration. We hypothesized that the access of microbes to different carbon pools in response to dry–rewet events occurs sequentially at different rates. We amended desiccated soils with ^13^C-labeled glucose and measured the rates of ^12^CO_2_ and ^13^CO_2_ respiration in real time after rewetting. Using these differentiated ^12^CO_2_ and ^13^CO_2_ respiration rate soils after rewetting, we were able to deduce when microbes are accessing different pools of carbon. Immediately upon rewetting, respiration of ^12^CO_2_ occurred first, with negligible ^13^CO_2_ respiration. Appreciable metabolism and respiration of the added ^13^C glucose did not occur until 15 min after rewetting. We conclude that, while all carbon pools are being accessed in the first 9 h after rewetting, the rate and timing at which new and existing carbon pools are being accessed varies. Within this study, using stable isotope-labeled substrates to discern which carbon pools are metabolized first uniquely illustrates how microorganisms access different carbon pools which has implications into understanding how carbon metabolism can further affect climate, carbon sequestration, and soil health.

## 1. Introduction

Rewetting of dry soil has a significant impact on soil organic carbon and nitrogen pools [1,2,3]. When soil has undergone a prolonged drying event or drought, the soil microbial community responds in several ways upon rewetting [4]. There is often a rapid increase in decomposition and mineralization of soil organic matter (SOM) over the first ½ hour to 24 h depending on the sampling timing, which declines within the first 4 weeks [5,6,7]. The more severe or prolonged the period of dryness, the more intense the responses [8]. For example, more N and carbon is released by soils exposed to dry–rewet cycles than by continuously wet soils [9]. Another common response is a disproportionate burst of respiration and a loss of soil carbon. These phenomena were first characterized by H.F. Birch half a century ago, and are termed the “Birch Effect” [2,10,11].

While the “Birch Effect” has been a topic of focus for many decades, the mechanisms and driving factors behind these responses are still unknown [12,13]. There are many hypotheses about the origin of the CO_2_, including both abiotic and biotic reactions. Abiotic release can originate from the displacement of CO_2_ trapped in soil pores [14] or the chemical release of CO_2_ from soil mineral surfaces [15]. Hypotheses contributing to the CO_2_ release from biotic origins can be separated into the metabolism of compounds that can be immediately accessed by the cell (e.g., carbon in intracellular or extracellular polymeric substances) or compounds that are not easily accessed by the cells (e.g., refractory extracellular DOC or dead microbial biomass (necromass) carbon). For simplicity, we will refer to the former as intracellular carbon and the latter as extracellular carbon. An example of intracellular carbon could be the rapid metabolism of accumulated osmolytes that are created to maintain osmotic pressure during dry periods upon rewetting. This rapid metabolism can prevent cell rupture by eliminating osmolytes from the interior of the cell [5,16]. Examples of extracellular carbon include dissolved organic carbon (DOC), plant biomass, exudates, microbial compounds, necromass [17,18], or the DOC from the depolymerization of extracellular metabolic products that have accumulated in the soil from extracellular enzyme activity [19]. While the CO_2_ respiration of extracellular carbon could occur by importing carbon into the cell and using it as energy via metabolism, the time frame for CO_2_ release from this carbon source would be slower than intracellular carbon. A third possible explanation for the Birch Effect could be a rapid increase in microbial biomass [20,21,22], for example, by growth on dead microbial cells that did not survive the dry down and rewetting process [23].

Across multiple moisture regimes, respiration has been seen to increase in the first 1–3 h after rewetting [3,5]. However, most research to date is based on single-time snapshots or through cumulative measurements (minutes, hours, or days) [24,25]. Warren (2016) found that withholding water from loam soil mesocosms for 21 days followed by a rewetting event resulted in a 5–10-fold increase in CO_2_ production within minutes, and respiration remained high for approximately 10 days using single time point measurements every thirty minutes [19]. Orchard et al. (1983) found similar results in a silt loam soil after injecting cumulative CO_2_ respired into a Li-Cor 1, 3, and 24 h after rewetting [26]. Cumulative respiration provides valuable information about soil respiration on longer time intervals; a fine scale rate of CO_2_ released provides information about the instantaneous microbial metabolism that drives cumulative respiration measurements. Weitz et al. (2020) showed that soil respiration responds almost immediately (within 1 min) upon rewet, and the peak rate of respiration could be missed with single time point measurements of cumulative respiration [27]. Microbial response to rewetting dry soil is extremely rapid, and the fine-scale mechanisms, including the source of respired carbon, are not well understood.

It has been shown that the bioavialibility of carbon is determined in part by moisture content, which influences the connectivity and transport of substrates and microbes [28,29,30,31]. As soils dry, the substrates that microbes use for energy, which are mostly water soluble, become dehydrated, concentrated, and possibly precipitate out [32]. Tecon has identified that water affects microbial dynamics in three fundamental ways; as a resource, as a solvent, and as a transport medium [33]. Upon rewetting of a desiccated soil, drought stress is released, and the water can redissolve and connect nutrients and microbes. For nutrients that are present in the cell during the dry down event, their consumption rate will be dependent on the rates of dissolution, transport, assimilation, and metabolism. Indeed, Liu et al. have proposed a model that differentiates between the rate of metabolism of soluble carbon versus dormant biomass, stating that the dormant biomass will be metabolized at a much slower rate [34]. Therefore, the rate at which metabolites inside the microbes are metabolized will not be subject to the nutrient transport or cellular transport steps and could be metabolized more rapidly than those metabolites that are extracellular in a dried or precipitated form. Additionally, those metabolites that are closely associated with the cell (captured in EPS and other extracellular structures) will only have to be imported into the cell using transporters compared to dried extracellular metabolites.

In this study, we aim to test if the rate and order of metabolism of different carbon pools are dependent on the differential rates of nutrient accessibility for microbial metabolism upon rewetting in desiccated soils. We examined three carbon pools: (1) background carbon, (2) immediately solubilized carbon, and (3) extracellular dried carbon along with an autoclaved abiotic sample. We used ^13^C-labeled glucose as a carbon source either dissolved in the rewetting solution to mimic immediately solubilized carbon and amended as a solid to the dry soil to mimic the extracellular dried carbon and compared these treatments to the unamended soil to mimic the carbon native to the soil. The rate of ^12^CO_2_ and ^13^CO_2_ production in the soils after rewetting was measured using the RTMS experimental setup where the rates of both isotopes of CO_2_ production were measured continuously for 6 h after rewetting in real time [35,36] and compared at different time points.

## 2. Methods

### 2.1. Field Site

Soil was collected from a field site managed by the Irrigated Agriculture Research and Extension Center (IAREC) at the Washington State University campus in Prosser, Washington (46°15′04″ N and 119°43′43″ W). This site is arid with an average precipitation of 9 inches a year [37]. The soil is a Warden sandy loam with a carbon content of 1.697% and pH 7.8 [38]. The average yearly temperature at this location is 12.06 °C with an average high of 18.5 °C, and an average summer high of 30.44 °C [37].

Soil was harvested in bulk sampling in October of 2018, sieved (4 mm) to homogenize and remove rocks and root material, and stored at 4 °C. The soil water holding capacity (WHC) was used to determine the mass of water to add to the rewetting experiments below. To determine total WHC, 5 g of soil were weighed onto a filter funnel, saturated with water, and drained for 2 h to determine wet soil mass. Subsequently, the saturated soil was weighed, then dried at 60 °C and reweighed until totally dry by mass to determine the mass of water held [7].

### 2.2. Incubations

Homogenized soil was partitioned into 12 incubation chambers (4 treatments × 3 replicates). For each experimental unit, 15 g dry weight equivalent of soil was weighed into Ball Mason jars (118 mL). The lids were modified with a gas-tight septum for a flow-through inlet and outlet fitted for the RTMS [27]. The jar headspace was optimized for the mass of soil by using sterile sand below a foil barrier. The soil samples were pre-incubated for 1 week and gradually exposed to increased temperatures from 10 °C until the target incubation temperature of 30 °C was reached. During this pre-incubation, samples were maintained at 75% WHC according to mass (15 g dry soil + 4.8 mL water). Once at 30 °C, WHC was no longer maintained, and soils were dried. After 6 days at 30 °C, moisture loss was confirmed based on gravimetric moisture content. Complete desiccation was validated on observation of a stable mass for 3 days. Subsequently, a 2-week drought incubation was initiated. Following the 2-week simulated drought, the soil incubations were rewetted to 75% WHC.

Soils were subjected to one of four experimental treatments. Control soils were rewet with 4.8 mL of autoclave-sterilized DI water (describe just as “water” below) to establish natural abundance ^12^CO_2_ and ^13^CO_2_ respiration from non-carbon-amended soils. The second treatment simulated DOC that is easily accessible to the cells upon rehydration and consisted of ^13^C universally labeled glucose dissolved in 4.8 mL of sterilized DI water (2.114 mg of ^13^C added as 1.03 mg of ^13^C glucose/mL of water, which matched the amount of carbon native to the soil). In the third amendment, the universally labeled ^13^C glucose was mixed into the soil as a powder (5.02 mg glucose for 2.114 mg of ^13^C) then rewet with 4.8 mL of sterilized DI water. This treatment was used to simulate the extracellular metabolites that were present in the soil during desiccation, then dried within the soil matrix and resolubilized after rewetting to be accessible to active cells. The fourth treatment was an abiotic treatment. Soils for the abiotic treatment were brought to 75% WHC, preincubated, and brought to 30 °C alongside the other incubation samples. Upon reaching 30 °C, the soils designated for abiotic treatment were sterilized via two cycles of autoclaving, with 24 h between each autoclave cycle. They were then returned to 30 °C, drought exposed for 2 weeks, and rewetted, identical to the water controls. While some microbial life may still exist, Carter et al. (2007) measured substrate-induced respiration and protease activity in autoclaved soils and saw a significant reduction compared to control soils, concluding that most microbial life is eliminated with autoclaving [39]. Berns et al. (2008) autoclaved soils and evaluated sterility by suspending soil in Ringer solution and evaluating growth after 21 days of incubation [40]. Lack of growth in the autoclaved soils was used to determine success in sterilization. These previous studies, along with others [41] have shown that autoclaved soil is considered sterile or diminished in microbial activity [41]. After drought, all soils were stirred with a spatula prior to rewetting with the above 4 treatments for consistency.

### 2.3. Soil Respiration Measurements

Dry (control), ^13^C liquid phase glucose-amended, ^13^C solid-phase glucose-amended and autoclaved soils were put into the incubation chambers, placed in line with the RTMS system, and flushed with CO_2_-free air for 12 h. Each sample was then rewet with sterilized DI water or glucose-amended sterilized DI water while being incubated at 30 °C. Soils were monitored for ^12^C- and ^13^C-CO_2_ for 24 h, using RTMS [27] via a Shimadzu QP2020 MS (Laval, QC, Canada) configured with our patented inlet manifold technology. Rates and flow paths were optimized for the soil vessel’s headspace volume and design. A constant 2.5 mL/minute flow rate was used to determine gas production in real time. CO_2_ standards were run each day to convert *m*/*z* intensities to ppm. This was achieved by running 3 L Tedlar gas bags containing known CO_2_ ppm concentration standards prepared from pure gas stocks (0, 1, 500, 1000, 2000, 5000 ppm CO_2_). The RTMS equipment collected 1440 min (24 h) of data per acquisition period at a scan rate of one scan every three seconds. Each data file of 86,400 scans was collected to monitor and calculate the soil sample responses as they occurred.

Through this experiment, we monitored respiration using RTMS for the simultaneous measurement of ^12^CO_2_ and ^13^CO_2_, at a temporal resolution of 3 s intervals through 6 h after the water addition. This granularity allows the continuous detection of both isotopic versions of CO_2_ respiration and can be separated into different time intervals. For this study, we chose the first 90 s, 0–15 min, 15–90 min, and up to 360 min.

### 2.4. Data Analysis

A linear model was fit to the 0 ppm CO_2_ standard and used to normalize the RTMS instrument background noise for each sample. Additionally, 50 scans before the sample water addition were used to standardize the baseline for each sample. A linear model was fit to carbon standards spanning a range of concentrations (see above) and used to convert from *m*/*z* MS peak height to carbon parts per million (ppm). The rate of carbon respired was determined by calculating the slope between every consecutive scan until the maximum peak intensity and taking the median of those slopes as representative of the entire sample. A fixed linear model and ANOVA (using the car package) was fit to test differences in the rate of response for the different carbon sources. All analyses were performed using the R programming language [42].

## 3. Results

The premise of these experiments is that, upon rewetting, the microbes will access carbon pools at different rates. Our experimental design aimed at testing the correlation of the rate and order of metabolism of different carbon pools with the differential rates of nutrient accessibility for microbial metabolism upon rewetting in desiccated soils. Resident background carbon is represented by our control soil treatment. Abiotic carbon is represented by our autoclaved soils where the microbial activity was reduced in those soils. Immediately soluble metabolites are represented by the solution carbon amendment where ^13^C glucose was dissolved in the rewetting solution. Completely dried metabolites were represented by a solid ^13^C glucose amended to the soils. Both ^13^C-amended samples targeted truly extracellular compounds, while the control sample sampled both intracellular and extracellular resident carbon. In the latter two samples, for the solution phase amendment, microbial access to the glucose was only subject to the diffusion and transport rates, while dried ^13^C glucose and native dried metabolites would be subject to dissolution, diffusion, and transport rates before microbial metabolism. By monitoring the ^13^CO_2_ respiration arising from the metabolism of ^13^C glucose, we were able to distinguish between metabolism of cellularly associated (intracellular and EPS associated) ^12^C and extracellular ^13^C.

To demonstrate that the ^12^CO_2_ release was the product of microbial activity, we compared CO_2_ respiration of autoclaved soil to live soil (Table 1). In the first 0–15 min after water addition, autoclaved soils produced CO_2_ at 49.31 ± 13.80 ppm/min compared to control soils with a rate of 83.28 ± 14.51 ppm/min, which is significantly less (1.68-fold) CO_2_ in the autoclaved soil (Table 1). While it has been shown that autoclaving soil can affect soil organic matter structure [40] and does not completely eliminate microbial activity [43,44], it does diminish microbial activity [39]. Our results indicate that, while there is abiotic CO_2_ release from the soils and potentially some microbial activity present after autoclaving, more than 50% of the CO_2_ arises from microbial respiration after water addition [39].

**Figure 1:**
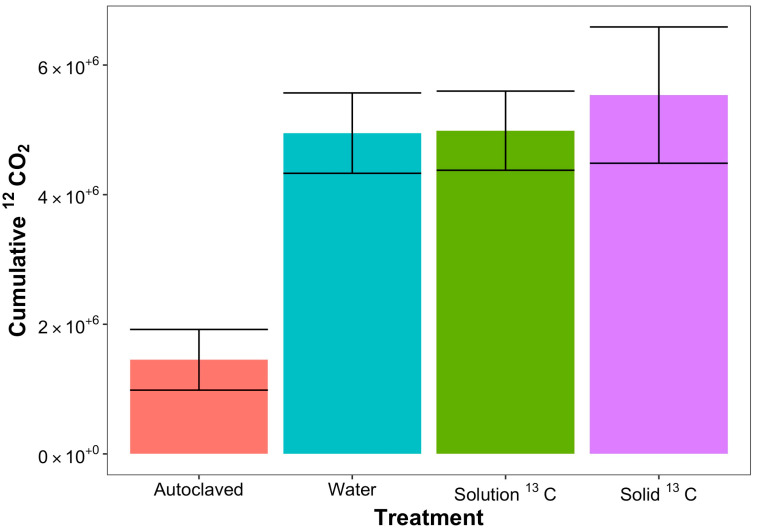
After 360 min, 6 h, cumulative ^12^CO_2_ respiration was consistent in all biotic (non-autoclaved) soil treatments. Sterile autoclaved soil produced significantly less CO_2_ over the course of the experiment (*p* < 0.05). While all other treatments produced approximately the same cumulative amount of CO_2_ (*p* > 0.1).

A comparison of the ^12^CO_2_ release from the four soils (dry (control), ^13^C liquid phase glucose-amended, ^13^C solid-phase glucose-amended, and autoclaved soils) revealed that the amount of ^12^CO_2_ released from the desiccated soils upon water amendment was independent of glucose amendment (liquid or solid) but dependent on whether the soil was autoclaved or not (Figure 1). The amount of ^12^CO_2_ released from the dry (control) soil, the ^13^C liquid phase glucose-amended, and the ^13^C solid-phase glucose-amended samples were statistically the same, while the amount of ^12^CO_2_ released from the autoclaved soils was reduced by 3-fold over the entire experiment. This result illustrates that the CO_2_ produced after water amendment mostly arises from microbial activity.

**Figure 2:**
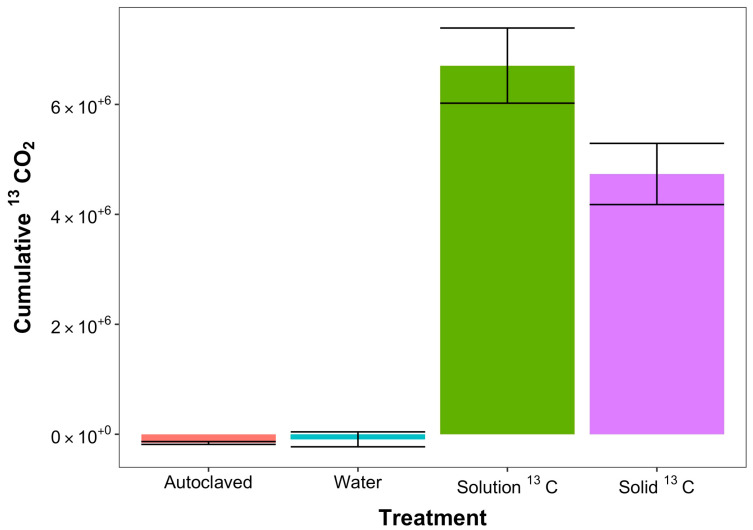
At the end of the experiment, 6 h, cumulative ^13^CO_2_ respiration remained negligible in native and autoclaved soil rewet with water, as expected. Samples treated with ^13^C glucose in solution respired a significantly greater amount of ^13^CO_2_ over the course of the experiment compared to solid ^13^C-treated samples (*p* < 0.05).

Since the ^13^CO_2_ could only arise from extracellular sources, to assess the relative contribution of extracellular carbon to the respiration pulse after desiccation–rewet events, we monitored the evolution of ^13^CO_2_ from the ^13^C liquid phase glucose-amended and ^13^C solid-phase glucose-amended soils. While the ^12^CO_2_ respiration was first detected at 63 s post water addition, the production of ^13^CO_2_ was not detected until 96 s and at a rate 51 times slower than ^12^CO_2_ production (Table 1). The cumulative ^13^CO_2_ and ^12^CO_2_ release from the four soil treatments over the entire 6-h time course of the experiment revealed very different results. (Figure 2). We observed 6.12 × 10^6^ ppm ^13^CO_2_ and 4.05 × 10^6^ ppm of ^13^CO_2_ produced from the solution-amended and solid-amended samples, less than 1000 ppm of ^13^CO_2_ was produced from the control and autoclaved samples. The solution-phase glucose released about 25% more ^13^CO_2_ in the first 6 h likely due to a slower rate of metabolism of the solid-phase glucose than the solution phase. Indeed, the peak production of ^13^CO_2_ from the solution-phase glucose-amended samples with an initial ^13^CO_2_ production rate of 6.49 ppm ^13^CO_2_/minute was reached at about 5 h at which time the production started to decline. Conversely, the initial rate of ^13^CO_2_ from the solid-phase samples was 3.7 ± 0.8 ppm ^13^CO_2_/min, and peak production was not reached until about 8 h.

In a closer look at the rate of ^12^CO_2_ production and ^13^CO_2_ production over shorter time scales, we compared the amount of ^12^CO_2_ and ^13^CO_2_ production in control treatments (water only) to those with solution or solid glucose additions at three time intervals: 0–15 min, 15–90 min, and 300–360 min (Table 1). In the first 0–15 min, the amount of ^12^CO_2_ produced from these three soil treatments were similar, with an average concentration of 2.68 × 10^5^ (±3.7 × 10^4^) ppm of CO_2_. As mentioned, ^12^CO_2_ respiration in the solution glucose treatment remained consistent with the control, and in 15–90 min, began to slow to an average rate of −0.65 ppm/^12^CO_2_/minute. In this study, the RTMS monitored respiration in real time and included a carrier gas that flowed through the mass spectrometer. This negative rate in respiration represents slowed respiration and the effect of dilution with the carrier gas, not an uptake of CO_2_.

^13^CO_2_ production from the labeled glucose treatments (solution and solid phase) was measurable during the first 15 min (Table 1). The average rate at which it was produced was 10 times slower than that of ^12^CO_2_ respiration (*p* < 0.05) in the same samples. This lower rate of ^13^CO_2_ respiration points to different carbon pools being accessed unequally, with the native soil carbon metabolized first. These results revealed that the soil microbes did not metabolize or respire the added ^13^C-labeled extracellular carbon source immediately following soil rewetting. The amount of ^13^CO_2_ produced from the control soil was negligible, as would be expected (1.31 ± 0.22 ppm CO_2_/min).

Results from the 15–90 min time frame revealed similar amounts of ^12^CO_2_ produced across the control, solution glucose, and solid glucose treatments (Table 1) and negligible ^13^CO_2_ from the water controls. In total, 5.5 × 10^5^ ± 5.5 × 10^4^ ppm ^13^CO_2_ at the rate of 6.49 ± 0.31 ppm ^13^CO_2_/minute was respired during this time frame from the solution glucose-treated sample, while 4.1 × 10^5^ ± 3.97 × 10^4^ ppm ^13^CO_2_ at the rate of 4.26 ± 0.24 ppm ^13^CO_2_/minute was respired during this time frame from the solution glucose-treated sample. This result indicates that the glucose added in solution was not assimilated by the cells for energy production until sometime after rewetting.

After 6 h of incubation (300–360 min), ^12^CO_2_ respiration remained consistent across all biotic samples. Unlike ^12^CO_2_, the ^13^CO_2_ respiration continued to increase in the solid ^13^C glucose-amended treatment (3.7 ± 0.8 ppm ^13^CO_2_/min). At this point, peak ^13^CO_2_ respiration in the ^13^C glucose solution treatment had already been reached, and the rate of respiration began to decline (−6.86 ppm ^13^CO_2_/min), indicating a decrease in the amount of ^13^CO_2_ respired in that sample.

**Figure 3:**
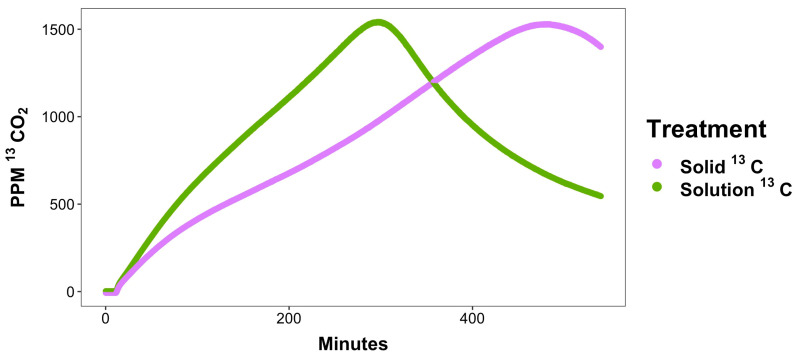
Time course representation of the ^13^CO_2_ production from the solution glucose-amended soil (green line) and the solid glucose-amended soil (purple line). Peak ^13^CO_2_ respiration in ^13^C glucose solution-treated samples occurred at an average of 290 min after rewet. ^13^CO_2_ respiration from samples treated with solid ^13^C glucose reached peak respiration at around 480 min.

As stated above, during the desiccation process, metabolites are dried or precipitated thus needing to be redissolved, diffused, and transported into the cell before they can be used for energy production. The comparison of our solution glucose to our solid glucose-amended samples reveals the significance of this dissolution rate to the overall microbial metabolic utilization rate. Solid ^13^C-labeled glucose soils generated 4.7 × 10^6^ ± 5.5 × 10^5^ ppm ^13^CO_2_ production over the course of the experiment; however, this occurred at a rate of 4.26 ppm ± 0.24 ppm ^13^CO_2_/minute. Compared to the overall ^13^CO_2_ production from the solution sample (cumulative of 6.7 × 10^6^ ± 6.8 × 10^5^ ppm at a rate of 6.49 ppm ^13^CO_2_/minute) the ^13^CO_2_ production was 1.5 times slower than the soluble glucose-treated soils. Indeed, the peak ^13^CO_2_ respiration in ^13^C glucose solution-treated samples occurred at an average of 290 min after rewet. ^13^CO_2_ respiration from samples treated with solid ^13^C glucose reached peak respiration at around 480 min (Figure 3). The delayed metabolism and respiration of the added ^13^C glucose in solid form continued through the remainder of the experiment, illustrating the delay in metabolite utilization caused by the need to dissolve dried metabolites in the rewetting solvent.

## 4. Discussion

For decades, soil respiration has been investigated as a key factor regulating soil carbon loss. Understanding the relative importance of different carbon pools is critical for managing ecosystem carbon balance in the face of changing environmental conditions. Drought rewetting is common, but the pathways leading to CO_2_ release remain uncertain. Here, we examined the relative importance and timing of three major pools of carbon that microbes respire during rewetting events; background carbon, immediately solubilized extracellular compounds, and completely dried and not readily solubilized extracellular compounds.

Throughout the entire experiment, there was an evolution of ^12^CO_2_ that arose from the metabolism of the background carbon. The use of the ^13^C glucose amendment allows us to follow the accession of immediately soluble metabolites (solution ^13^C glucose) and dried metabolites (solid ^13^C glucose). The typical burst of ^12^CO_2_ that was described by Birch over a century ago, which occurred in the first 15 min after rewetting of our desiccated soils, was followed by a decrease in ^12^CO_2_ respiration until a steady state was reached. The similarity of the ^12^CO_2_ release within the first 15 min in the three biotic samples suggests that the first pool of carbon that the microbes are able to access are not from the easily soluble pool (represented by the solution sample) but arises from native carbon that is already closely associated with the microbial cells. Glucose is easily accessible and a known osmolyte and root exudate [45,46]. Since the ^13^C from the glucose dissolved in the rewetting solution is immediately available to the cells (subject to only the diffusion and transport rates), the fact that the rate of ^13^CO_2_ production in the first 15 min is 10× lower than that of the ^12^CO_2_ production in the solution glucose-amended soils suggests that this pool is not representative of extracellular metabolites that do not have to be redissolved before diffusion and transport. We hypothesize that given the extremely rapid rate of metabolism of these compounds (arising in the first 90 s after rewet) that the rate of diffusion and transport are also small, and these compounds are either already inside the cell, or in the area directly surrounding the cell (possibly stored in the EPS) to enable immediate utilization. Warren et al. have suggested that, during dry down, osmolytic compounds are synthesized and stored in the cell to protect against the effects of desiccation [3]. Upon rewetting, it has been suggested that these compounds need to be liquidated from the cell by either export or metabolism [3], and we believe the rapid ^12^C signature is due to this rapid metabolism of these intracellular osmolytes.

The decrease in the ^12^CO_2_ production after the first 15 min with a concomitant increase in the ^13^CO_2_ production suggests that, within this time frame, the microbes have started assimilating the extracellular forms of carbon. The increased rate of metabolism of the solution-phase glucose over the solid-phase glucose indicates that the solubilization of dried extracellular carbon plays a role in the rate at which microbes can assimilate this carbon pool. Additionally, the difference of 190 min between the peak utilization of carbon from the solution-phase glucose versus the solid-phase glucose also highlights the difference in the rate at which the microbes access the different pools of carbon. Manzoni has created a conceptualized model of carbon utilization for both first-order and dormancy carbon pools [47]. Lui extends this model to allow for changes in the biomass response to differential metabolite solubility and saturation [34]. We believe that the differential rates of carbon utilization between the solution-phase glucose and the solid-phase glucose supports this dormancy model of carbon utilization in soils.

Finally, Warren et al. (2020) results suggested that osmolyte concentrations increased with soil drought, and upon rewet, osmolytes were not only exported into the extracellular matrix [3], but were also responsible for the rapid increase in respiration seen upon rewet. Slessarev et al. (2020) also examined how extracellular carbon and cellular carbon contribute to the increased respiration seen when drought-exposed soils are rewet. In soils spanning various geographical sites across California, after 6 weeks of drought exposure, CO_2_ respiration was measured at 3 and 6 h after rewetting [48]. Their results showed a strong correlation between biomass and respiration, as well as a decrease in the osmolyte trehalose within biomass over the incubation, though trehalose concentration within the water extractable fraction of their soils was below detection. This result paired with our respiration results suggests that this “lost trehalose” was not exported, but rather was metabolized and respired upon rewet.

However, Warren et al. also suggest that this metabolism arises also from the turnover and metabolism of osmolytes exported by neighboring cells [3,19]. These osmolytes, however, are subject to the export, diffusion, import, and metabolic rates within the soils, which in most studies are measured in terms of minutes or hours at best [46,47,48]. In our studies, we resolved the difference in ^12^CO_2_ vs. ^13^CO_2_ production to the order of seconds, where we find ^12^CO_2_ is produced earlier (initiating at 60 sec at a rate of 66.89 ± 12.05) than the ^13^CO_2_ (initiating at 96 s at a rate of 7.86 ± 0.55). These findings using the ^13^C solution-phase glucose suggest that the sharing of osmolytes is less likely since these compounds are subject to rates of export, diffusion, and import to the rapidly metabolizing cell (usually measured on the minutes-to-hours time frame) [47]. We were able to distinguish the production between ^12^CO_2_ and ^13^CO_2_ at such a high temporal granularity (every 2 s) immediately following the rewetting of the soils, and because the solution-based ^13^C glucose was already dissolved in the rewetting solution. Similar to the postulate put forward in the model by Liu et al., where, through a solubilization process, this complex carbon is converted to soluble carbon, which is considered bioavailable for respiration and production of CO_2_, the already solubilized carbon is only subject to the rates of diffusion and import before it is available to be metabolized [34,47]. For this paradigm of osmolyte sharing contributing to the initial burst of CO_2_ from the microbes to hold true, the ^13^CO_2_ and the ^12^CO_2_ production would have to be the same in this system given that the amount of ^13^C glucose added was at least half of the resident carbon in the cell. For this reason, our hypothesis that only the metabolism of compounds that are intracellular or in the immediate space around the microbes can be responsible for the immediate burst of CO_2,_ as seen in our experiments, and that the other carbon pools are accessed at later times.

## 5. Conclusions

Our results suggest that the disproportional amount of CO_2_ released upon rewet, known as the Birch effect, may be due to the metabolism and respiration of carbon already associated with the cells, possibly accumulated during dry down. Our data show that the immediate burst of CO_2_ seen in the first 15 min arises from native carbon, but, considering the rates needed for dissolution, diffusion, and cellular transport of the carbon in the soils, it is most likely that this carbon arises from compounds that are intracellular or in the immediate environment of the microbes. We have also shown that the dissolution rate of metabolites in the soil has a great effect on the rate at which these compounds are made available and metabolized by the soil microbes, thus adding support for the dormancy model of carbon utilization in soils. Finally, we believe that we have shown that an increased granularity of measurements reveals a finer scale view of the molecular mechanisms of the “Birch Effect” in soil systems.

## Figures and Tables

**Table 1:** ^12^C and ^13^CO_2_ respiration rate and cumulative respiration for 0–15, 15–90, or 300–360 min after rewetting for all 4 treatments (autoclaved soil, native soil with water, ^13^C glucose solution, and ^13^C solid glucose).

	^12^CO_2_			
	Autoclaved	Water	Solution ^13^C	Solid ^13^C
Rate: ppm CO_2_/min: 0–15 min	49.31 ± 13.80	83.28 ± 14.51	66.89 ± 12.05	84.16 ± 15.36
Rate: ppm CO_2_/min: 15–90 min	−5.91 ± 1.94	−4.42 ± 2.15	−0.95 ± 1.57	−3.46 ± 1.72
Rate: ppm CO_2_/min: 300–360 min	−0.19 ± 0.10	−0.29 ± 0.21	−0.66 ± 0.05	−0.05 ± 0.29
Cumulative: ppm CO_2_: 0–15 min	180,910.4 ± 54,105.69	303,464.3 ± 58,168.41	233,231.7 ± 50,054.46	303,105.2 ± 58,366.17
Cumulative: ppm CO_2_: 15–90 min	696,171.9 ± 181,511.2	1,563,636 ± 171,505.3	1,453,573 ± 164,849.5	1,673,091 ± 228,794.6
Cumulative: ppm CO_2_: 300–360 min	60,161.04 ± 19,173.33	555,203.4 ± 90,076.06	592,211.3 ± 31,433.3	670,661.1 ± 90,516.3
	**^13^CO_2_**			
	**Autoclaved**	**Water**	**Solution ^13^C**	**Solid ^13^C**
Rate: ppm CO_2_/min: 0–15 min	0.85 ± 0.21	1.31± 0.22	7.86 ± 0.55	6.85 ± 0.57
Rate: ppm CO_2_/min: 15–90 min	−0.12 ± 0.02	−0.16 ± 0.08	6.49 ± 0.31	4.26 ± 0.24
Rate: ppm CO_2_/min: 300–360 min	−0.04 ± 0.02	−0.03 ± 0.03	−6.86 ± 0.96	3.69 ± 0.80
Cumulative: ppm CO_2_: 0–15 min	−3542.3 ± 1102.07	1047.22 ± 5920.18	15,962.34 ± 5920.18	14,631.52 ± 3971.39
Cumulative: ppm CO_2_: 15–90 min	−21,799.99 ± 5225	−142.2 ± 7872.39	550,644.1 ± 55,425.45	408,071.2 ± 34,657.65
Cumulative: ppm CO_2_: 300–360 min	−33,876.62 ± 3446.91	−28,168.73 ± 21,098.52	1,552,464 ± 62,269.63	1,362,448 ± 103,241.9

## Data Availability

EMSL supports the FAIR data principles and encourages data reuse. Data is made available through EMSL’s data portal (https://search.emsl.pnnl.gov/) where data refers to sample metadata, raw instrument data, associated experiment metadata, and processed data, and is released to the public in open access formats.
